# Increasing predation risk with light reduces speed, exploration and visit duration of invasive ship rats (*Rattus rattus*)

**DOI:** 10.1038/s41598-019-39711-3

**Published:** 2019-03-06

**Authors:** Bridgette Farnworth, Richard Meitern, John Innes, Joseph R. Waas

**Affiliations:** 1 Biological Sciences, School of Science, University of Waikato, Private Bag, 3105 Hamilton, New Zealand; 2 University of Tartu, Department of Zoology, Institute of Ecology and Earth Sciences, Vanemuise 46, 51014 Tartu, Estonia; 3 Manaaki Whenua – Landcare Research, Private Bag 3127, Hamilton, 3240 New Zealand

## Abstract

Exploiting predation cues to deter pests remains an untapped management tool for conservationists. We examined foraging and movement patterns of 20 wild ship rats *(Rattus rattus)* within a large, outdoor ‘U maze’ that was either illuminated or dark to assess if light (an indirect predation cue) could deter rodents from ecologically vulnerable locations. Light did not alter rats’ foraging behaviour (latency to approach seed tray, visits to seed tray, time per visit to seed tray, total foraging duration, foraging rate) within the experimental resource patch but three of seven movement behaviours were significantly impaired (53% fewer visits to the maze, 70% less exploration within the maze, 40% slower movement within the maze). The total time males spent exposed to illumination also declined by 45 minutes per night, unlike females. Individual visits tended to be longer under illumination, but the latency to visit and the latency to cross through the U maze were unaffected by illumination. Elevating predation risk with illumination may be a useful pest management technique for reducing ship rat activity, particularly in island ecosystems where controlling mammalian predators is paramount to preserving biodiversity.

## Introduction

Controlling introduced mammalian pests is central to conserving biodiversity in island communities^1^ such as New Zealand^2^, Hawaii^3^ and New Caledonia^4^. For example, New Zealand’s “Predator Free 2050” campaign requires the development of tools that are more efficient than the single-capture traps and broad-spectrum anticoagulants established decades ago^2,5,6^. Understanding a pest’s behavioural ecology may be critical for the successful development of new lures, baits and deterrents, and may enable conservationists to control or eradicate pests by altering activity patterns^7,8^. Manipulating predation risk by applying ‘predation cues’ is an innovative pest management strategy for ecologically sensitive areas^9–13^. The ‘risk allocation hypothesis’^14^ suggests that prey align the timing of their activities with their perception of danger. Predation risk is conveyed by (1) direct (e.g. a predator’s visual^15^, tactile^16^, olfactory^17,18^ or auditory attributes^19^) or (2) indirect predation cues (e.g. microhabitat structure^20^ or light levels^21^).

Ambient light is an indirect cue of predation risk because it can improve the visual acuity of predators and makes prey movement conspicuous^21,22^. Sensitivity towards illumination allows prey to behave flexibly and avoid unsafe foraging patches; for example, rodents reduce activity when exposed to moonlight (e.g. house mice *Mus musculus*^13^; Allenby’s gerbils *Gerbilus andersoni allenbyi*^23^; kangaroo rats *Dipodomys*^24^; deer mice *Peromyscus maniculatus*^21^) and artificial light (e.g. white footed mice *Peromyscus leucopus*^25^; wood mice *Apodemus sylvaticus*^26^; house mice^9^; Cape York rat *Rattus leucopus*^27^; bush rat *R. fuscipes*^27^). While New Zealand has no native species of rodent, the four introduced species (*M. musculus, R. rattus, R. norvegicus, R. exulans*) significantly damage native flora and fauna (reviewed by^28^); for example, rodents reduce the reproductive success of native birds through nest predation and resource competition^29–32^. To protect endemic fauna and flora, barriers of light may effectively deter rodents from accessing New Zealand’s valuable conservation estates, such as eco-sanctuaries^9^.

Eco-sanctuaries provide significant conservation benefits^33^ and are predicted to play a key role in achieving New Zealand’s predator free vision^5^. Pest fences are often erected at eco-sanctuaries to minimise reinvasion after eradicating mammals^34,35^. Ship rats and house mice threaten the ecological integrity of fenced eco-sanctuaries though, as rodents rapidly locate breaches in eco-sanctuary fencing while travelling along the fence base or within the gutter of the fence hood^35^. Illuminating sections of fencing may deter rodents from accessing vulnerable sites, such as storm-damaged fencing or exposed coastal interfaces, where fencing only partially extends across peninsulas^9^.

Light could offer a novel solution for manipulating pest behaviour; however, while light deters rodents from artificial food patches^9,13,25,27^, previous studies have not explored if illumination supresses rodent movement (i.e. prey responses may vary across contexts). For example, feeding individuals may experience greater risk than moving individuals because animals can be vigilant while exploring^36^ but handling complex food items diverts a forager’s attention from approaching predators^37–40^. Additionally, male and female rats may not respond identically to risk. Female rats invest heavily in parental care and may respond more cautiously towards predation cues than promiscuous male rats^41^. Males may trade off exposure to risk for additional mating opportunities or better foraging prospects^41^, especially during the breeding season when they travel across large home ranges^42–44^.

In this study, we add to existing literature on both predation cues and pest control techniques, by exploring the efficacy of applying light to manage pest activities. We assessed wild rodents’ perception of predation risk by investigating if ship rats avoided illuminated areas. We also examined if artificial illumination influenced foraging and movement patterns differentially, and if a rat’s sex influenced responses. We predicted that ship rats would minimise the number of visits to an artificially illuminated chamber and spend less time exposed to the light. We expected that rats would decrease the number of foraging visits, total feeding duration and foraging rates under illumination; we also predicted that, when exposed to light, females might be more sensitive to predation cues than males. Our findings identify contexts where the careful application of artificial light may provide conservationists with a behaviourally-based management solution (see^45^) for protecting biodiversity within natural ecosystems.

## Methods

All experiments were performed in accordance with relevant guidelines and regulations that were approved by the University of Waikato (Protocol 992) and the Lincoln University Animal Ethics Committees (Application No. 655).

### Animal housing

Twenty wild rats (10 male [average weight: 159 g ± 38]/10 female [average weight: 115 g ± 19]) were caught in live capture traps from native forests in Canterbury, New Zealand over a four week period (20 July to 17 August 2016). On arrival at Lincoln University’s research facility (Lincoln, New Zealand), rats were weighed, treated for parasites, sexed and housed individually within cages measuring 48 × 30 × 20 cm high. Housing cages were lined with wood shavings and contained food (i.e. a mix of standard lab chow pellets, fresh fruit/vegetables and seed mix), water, nesting material and enrichment (e.g. cardboard tunnels and boxes). Rats were kept under natural lighting conditions and at ambient temperatures by placing their individual housing cages in an outdoor enclosure that was positioned in a sheltered area of the research compound. All animals were allowed at least ten days to acclimatise to their cages and feeding regime before they were used in trials.

### Experimental design

**Figure 1:**
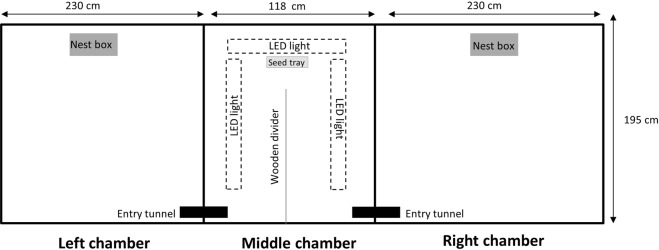
Configuration of the test pen showing how the middle chamber incorporated a ‘U maze’ with a wooden divider and contained a seed tray placed under three LED lights, which enabled the level of illumination to be adjusted (high: c. 1000 lux; low: <1 lux).

The test pen was situated outdoors within the research compound and comprised of three chambers: left, middle, and right, covered by a fine mesh rendering them escape proof (Fig. 1). The left and right chambers had bark chip flooring and plywood covering the roof to reduce moonlight as a confounding variable. A wooden nest box (51 × 32 × 30 cm high) containing fresh bedding material and water was available in the right and left chambers (Fig. 1).

The middle chamber also had bark chip flooring but both ceiling and walls were plywood. A wooden divider (150 × 80 cm high) ran two thirds of the way through the centre of the chamber, creating a ‘U maze’ between the entry tunnels from the left and right chambers into the middle chamber; a seed tray was positioned opposite the divider (Fig. 1). The entry tunnels were also fashioned from plywood (31 × 10 × 10 cm high) and were the only points of entry to the middle chamber. Within the middle chamber, three LED lights (one above the seed tray measuring 75 × 7 × 6 cm high, 19 watts, 220–240 volts; and two over the arms of the ‘U’ maze measuring 135 × 7 × 6 cm high, 41 watts, 220–240 volts; Planox Eco, Rudolf Zimmermann, Bamburg, Germany) were suspended from the ceiling (150 cm above the seed tray surface and 88 cm above the surface of each arm) so that light projected at an intensity of c. 1000 lux onto the floor of the middle chamber.

Two infra-red cameras (Techview QV3034 system, 3.6 mm lens; Jaycar Electronics, Auckland) were angled to capture the floor of the middle chamber, giving a bird’s eye view of any rodent activity that occurred within the U maze and seed tray. The seed tray (20 × 16 × 8 cm high) contained 1150 g of sand (c. 4 cm deep) mixed with 100 unhusked sunflower seeds (total weight <3 g) and was used to determine how motivated rats were to obtain a reward under two conditions: light (= high predation risk) and dark (= low predation risk). Counting the number of food items remaining in a foraging patch (Giving-Up Density or GUD)^46^ is commonly done to investigate the impact of predation risk on the foraging behaviour of small mammals^9,13^ and can be used as a ‘behavioural indicator’ of patch use^47^. During the two evenings directly preceding a test, subjects were presented with a clean seed tray in their housing cage that also contained 100 unhusked sunflower seeds, to familiarise them with the feeding apparatus. Seeds were counted the following day to establish that seeds were being removed regularly from the tray; if no seeds were taken, peanut butter was smeared along the side of the tray using a gloved finger until rats foraged within the seed tray for two nights without the peanut butter bait.

### Procedures

On each test night (10–30 August, 2016; Austral winter), one rat (food deprived for 12 hours) was transported within its housing cage to the test pen at dusk. The housing cage was placed on its side within either the left or right chamber, with the top opened to allow the rat to exit and explore. The rat was able to explore both right and left chambers by travelling through the smaller middle chamber; it was also freely able to forage at the seed tray within the middle chamber *ad libitum*. The middle chamber exposed subjects to their allocated treatment. The chamber (left vs. right) that a rat was released into was randomly assigned, while the order of treatments was systematically assigned according to sex. Tests were blocked so that 10 animals (five males; five females) experienced illumination and another 10 animals (five males; five females) experienced darkness. Animals were only tested once and were naïve to the test pen. Each test began once the subject’s housing cage was opened and ended the following morning when the rat was recaptured by closing it in the nest box and returning it to its home cage.

After each test the bark flooring within all chambers of the test pen was hosed with water, raked throughout the pen to evenly redistribute it, and left to dry. Nest boxes from the left and right chambers were hosed with water and left to dry outdoors, while fresh bedding material was placed into clean nest boxes for the next test. Seed trays were cleaned with hot water and detergent, dried and refilled. We reviewed video footage using a high resolution computer screen (Dell Optiplex 9010; 1920 × 1080 p resolution).

We recorded the following ‘movement’ behaviours: (a) latency to enter the middle chamber; (b) number of visits to the middle chamber; (c) amount of time spent per visit to the middle chamber; (d) total amount of time spent in the middle chamber; (e) rate of movement within the middle chamber (i.e. number of pixels moved per second); (f) latency to the first crossing from the left chamber to the right (or vice versa); and (g) the number of crossings from the right chamber through to the left chamber (or vice versa).

We also recorded the following ‘foraging’ behaviours: (a) the latency to come into contact with the seed tray; (b) the number of visits to the seed tray; (c) the amount of time per visit to the seed tray; (d) the total amount of time spent foraging at the seed tray; and (e) foraging rate (i.e. seeds remaining/Giving-Up Density).

### Video analysis

Rat movement in recorded videos was analysed using custom animal tracking software, which was designed using Python 2.7 with SciPy^48^ and OpenCV 3.0.0 libraries^49^. The background subtraction, used for object tracking, implemented a k-nearest neighbours algorithm^50^. For more accurate results the size and colour of the moving rat was included in noise filtering. The raw tracking data were processed in Excel (Windows 2007) to extract each of the univariate response variables described above.

### Statistical analysis

The data from one rat was excluded due to abnormal behaviour (i.e. excessive delay to enter the middle chamber and an extremely low foraging rate) compared with all other rats within the same treatment cohort so statistical analyses were conducted on 19 rats (5 males in darkness; 5 males in illumination; 4 females in darkness; 5 females in illumination). We used a PERMANOVA (PRIMER 7) to determine treatment effects on each univariate response variable using Euclidean distance matrices. The analysis included treatment (2 levels) and sex (2 levels) as fixed factors.

## Results

### Movement

Light significantly reduced both the number of visits to the middle chamber (by 53%) and the number of crossings between end chambers (by 70%; Table 1). While light did not prevent rats from crossing into the opposing chamber (nine out of the ten rats crossed), rats significantly reduced their rate of movement when the chamber was illuminated (by 40%). Reducing movement may have also increased the duration of each visit to the illuminated chamber because rats took twice as long to end a given visit in light than in dark, a difference which approached significance (p = 0.07). A significant Sex x Treatment effect occurred for the total time spent in the middle chamber: males reduced the total time spent within the illuminated middle chamber (by 45 minutes; t = 2.44, p = 0.05) while the total times for females did not differ across treatments (t = 1.11, p = 0.34). Light did not influence the latency to enter the middle chamber or the latency to cross the middle chamber (Table 1).

**Table 1:** Summary of PERMANOVA results (Euclidean distance) on univariate measures of ship rat movement behaviour for light and dark treatments with significant effects (*p* ≤ 0.05) indicated in bold.

Response variable	Treatment	Mean (±95% CI)	Term	df	pseudo-F value	*p*
Latency to enter mid chamber (min)	Light (n = 10)	33.2 (±16)	Sex × Treatment	1	0.04	0.85
	Dark (n = 9)	44.0 (±30)	Sex	1	1.39	0.274
			Treatment	1	0.44	0.54
Mid chamber visits	Light (n = 10)	101.0 (±37)	Sex × Treatment	1	0.58	0.51
	Dark (n = 9)	215.0 (±82)	Sex	1	0.21	0.71
			Treatment	1	5.77	0.01*
Time per visit to mid chamber (sec)	Light (n = 10)	91.2 (±62)	Sex × Treatment	1	1.03	0.39
	Dark (n = 9)	33.8 (±8)	Sex	1	0.64	0.55
			Treatment	1	2.90	0.07
Duration within mid chamber (min)	Light (♀ n = 5; ♂ n = 5)	Female: 133.2 (±61) Male: 78.2 (±27)	Sex × Treatment	1	4.49	0.04*
	Dark (♀ n = 4; ♂ n = 5)	Female: 91.2 (±31) Male: 121.8 (±23)	Sex	1	0.36	0.61
			Treatment	1	<0.01	0.97
Rate of movement (pixels per second)	Light (n = 10)	41.4 (±7)	Sex × Treatment	1	0.01	0.91
	Dark (n = 9)	69.4 (±19)	Sex	1	1.62	0.23
			Treatment	1	7.20	0.02*
Latency to cross mid chamber (min)	Light (n = 9)	166.2 (±122)	Sex × Treatment	1	0.14	0.85
	Dark (n = 9)	160.1 (±114)	Sex	1	0.15	0.85
			Treatment	1	1.64	0.23
Mid chamber crossings	Light (n = 10)	56.1 (±35)	Sex × Treatment	1	0.40	0.59
	Dark (n = 9)	169.8 (±77)	Sex	1	0.17	0.73
			Treatment	1	6.98	<0.01*

### Foraging

None of the foraging behaviours differed significantly across treatments (Table 2) but in accordance with our predictions rats tended to reduce their total foraging time within the seed tray, make fewer visits to the seed tray and have lower foraging rates when the middle chamber was lit. Under illumination, rats also tended to approach the seed tray faster and stay for longer per visit to the seed tray compared with the dark treatment (Table 2).

**Table 2:** Summary of PERMANOVA results (Euclidean distance) on univariate measures of ship rat foraging behaviour for light and dark treatments.

Response variable	Treatment	Mean (n = 19) (± 95% CI)	Term	df	pseudo-F value	*p*
Latency to approach seed tray (min)	Light (n = 10)	128.4 (±95)	Sex × Treatment	1	0.14	0.85
	Dark (n = 9)	131.9 (±99)	Sex	1	0.15	0.84
			Treatment	1	1.64	0.24
Visits to seed tray	Light (n = 10)	14.7 (±5)	Sex × Treatment	1	1.68	0.21
	Dark (n = 9)	18.3 (±6)	Sex	1	0.00	0.95
			Treatment	1	0.67	0.42
Time per visit to seed tray (sec)	Light (n = 10)	113.3 (±45)	Sex × Treatment	1	0.13	0.73
	Dark (n = 9)	100.3 (±24)	Sex	1	0.81	0.38
			Treatment	1	0.18	0.67
Total foraging duration at seed tray (min)	Light (n = 10)	22.2 (±5)	Sex × Treatment	1	0.25	0.79
	Dark (n = 9)	26.3 (±5)	Sex	1	0.30	0.74
			Treatment	1	1.89	0.18
Foraging rate (seeds remaining/GUD)	Light (n = 10)	6.5 (±6)	Sex × Treatment	1	1.96	0.18
	Dark (n = 9)	4.9 (±4)	Sex	1	0.05	0.84
			Treatment	1	0.15	0.72

## Discussion

### The effect of illumination on movement and foraging behaviours

To examine if predation cues altered movement and foraging behaviours differentially, we examined activities associated with (a) exploration of the maze and (b) accessing food, under illumination (high predation risk) and in darkness (low predation risk). Ship rats changed their movements according to the level of risk by visiting the middle chamber less and reducing their use of the chamber as a thoroughfare under illumination; they also slowed their movement within the chamber under illumination, perhaps to remain inconspicuous and minimise detection by predators. The significant reduction in exploration under illumination suggests that, like other rodent species, ship rats associate artificial illumination with a greater risk of capture by their nocturnal predators (e.g. feral cats [*Felis catus*], mustelids [*Mustela erminea, M. putorius furo, M. nivalis*])^42^. However, not all movement behaviours were affected by illumination. For example, light did not delay rats from entering the middle chamber or from crossing into the opposing chamber. In these instances, illumination may have extended ship rats’ perceptual range and increased their spatial awareness of the middle chamber^51^. For our maze-naïve subjects, rapidly gaining an awareness of their surroundings would be advantageous, as transient individuals experience greater predation risk than those already familiar with their environment^52^.

We expected foraging activities to decrease under illumination, but no differences occurred across light and dark treatments. The design of our seed tray may not have produced diminishing returns^53^ and therefore the foraging rates recorded (including four instances where rats consumed all available seeds) may not accurately represent how light influences feeding behaviour of ship rats (especially given our small sample size). In addition, our maze only provided one exposed foraging patch and the inability to avoid risk may have caused rats to alter their foraging strategies. For example, on average, rats made fewer visits to the illuminated seed tray and spent less total time foraging, yet they consumed an equal volume of seeds across both treatments. Feeding under risk may have been profitable if rats obtained rewards faster by utilising light to forage more efficiently. Energetic state may have further contributed to rats’ foraging decisions, as energetically stressed animals accept a higher risk of predation while feeding^54^. In our study, rats were food deprived and experienced low overnight temperatures (average = 6.8 °C) that may have imposed additional thermoregulation costs^55^, leading them to exploit the food source regardless of the level of predation risk. Hungry rodents may abandon ‘avoidance tactics’ when caloric benefits are greater than the risk of being caught. While avoidance is the primary mechanism for reducing predation, if starvation risk forces prey into areas with high predation risk, animals may prioritise behaviours (e.g. early threat detection and rapid responses to danger^56^) that allow prey to exploit foraging patches with higher predation risk^57^ but minimise the probability of capture.

### Sex differences in behaviour

Foraging behaviours did not differ across sexes. However, light had opposing effects on the amount of time spent in the central chamber – females tended to spend more time in the central chamber under illumination (but not significantly so), while males spent significantly less time there under illumination. Male and female ship rats are unlikely to show dichotomous behaviour due to sexual dimorphisms – males and females show little variation in colouration, weaponry, ornamentation, or size – they are equally conspicuous under illumination. However, males hold larger home ranges than females^58^ and may therefore encounter predators more often^59^ potentially selecting for more cautious movement. Females hold smaller ranges but may acquire a more detailed knowledge of that range^60^, perhaps in preparation for the breeding season where they are more sedentary^61^ and reduced mobility requires them to have precise information on local foraging, denning and sheltering opportunities; the need for local knowledge may have led non-breeding females to exploit light as a way of rapidly advancing their knowledge of a local but unfamiliar patch of habitat. The female strategy may change once she is pregnant (i.e. heavier and less agile), due to greater predation risk^62^. Further research on seasonal changes in antipredator behaviour would be useful.

### Implications for management

Nine out of ten ship rats crossed the middle chamber under illumination, indicating that light did not act as a ‘virtual barrier’ under the conditions of our experiment. We acknowledge that our sample size may have limited our statistical power to detect all significant differences across movement and foraging behaviours; nevertheless our results demonstrate that light significantly reduced visits and exploration through an unfamiliar area, which indicates that a wider barrier of illumination may substantially impact rodent movement. Biological control using predators can be effective (e.g. meat ants *Iridomyrmex reburrus* reduce the abundance of invasive cane toads, *Bufo marinus*^63^) but predation cues alone, trialled for conservation management within island (e.g. New Zealand^13,64^) and mainland ecosystems (e.g. United States^12^), have rarely demonstrated success in the field. Based on our results, the efficacy of light to control pest activities deserves further attention within Ecologically-based Rodent Management (EBRM) practises^65^. For example, light could be used in combination with other EBRM deterrents (e.g. plant secondary metabolites^66^) or control mechanisms (e.g. habitat manipulation^65,67^). Utilising light in conjunction with trapping and baiting strategies may also reduce rodent reinvasion at pest-free eco-sanctuaries or at unfenced conservation estates that receive predator control. Influencing the behavioural responses of rodents could be instrumental for rodent control if conservation managers can create conditions where rodents make disadvantageous decisions^67^.

While light presents significant opportunities for rodent management^9,27^, conservationists must also prudently consider any negative effects of illuminating fragile ecosystems. We found discrepancies between male and female behaviour that could impact the way light is used as a management strategy. For example, females may be less likely to avoid illuminated areas, which could lead to sex-biased invasion risk when protecting damaged eco-sanctuary fencing. Additionally, circadian behaviours (e.g. singing, foraging, diel movements and sleep) and seasonal biological events (e.g. growth, reproduction, migration, flowering and leaf loss) of non-target flora and fauna would be affected by nocturnal lighting (reviewed in^68^). Light-sensitive native species could change behaviour patterns or distributions to the detriment of local populations (e.g. New Zealand tree [*Hemideina thoracica*] and cave weta [Family *Rhaphidophoridae*]^10^). Mitigation strategies, such as (i) reducing lighting duration, (ii) minimising light ‘trespass’ into unintended areas and (iii) using an appropriate intensity and spectral composition could reduce negative ecological consequences^69^.

## Conclusion

Incorporating illumination as an indirect predation cue to elevate risk for invasive ship rats may be a useful strategy for controlling their activities at specific local sites (e.g. pest fence breaches) or at larger scales (e.g. on docks of pest free islands or at the land-sea interface of fenced peninsulas). Using risk perception to elicit avoidance responses in the context of pest management illustrates how behavioural ecology can contribute to species conservation through the creation of novel management strategies.

## Data Availability

The datasets generated during and/or analysed during the current study are available from the corresponding author on reasonable request.
